# Supplementary material for: Pyruvate Dehydrogenase Kinase 1 inhibition mediated oxidative phosphorylation enhancement in cartilage promotes osteoarthritis progression
Source: BMC Musculoskelet Disord. 2023 Jul 20;24:597. doi: 10.1186/s12891-023-06585-6 (PMC10357736; doi:10.1186/s12891-023-06585-6)
Supplement: Supplementary file 1 — Additional file 1. [file 12891_2023_6585_MOESM1_ESM.zip › BP.Gsea.1653623667859/GOBP_RIBONUCLEOPROTEIN_COMPLEX_SUBUNIT_ORGANIZATION.html]

Details for gene set GOBP\_RIBONUCLEOPROTEIN\_COMPLEX\_SUBUNIT\_ORGANIZATION[GSEA]

|  || Dataset | 55457\_collapsed\_to\_symbols.cls.cls #OA\_versus\_normal.cls.cls #OA\_versus\_normal\_repos |
| Phenotype | cls.cls#OA\_versus\_normal\_repos |
| Upregulated in class | normal |
| GeneSet | GOBP\_RIBONUCLEOPROTEIN\_COMPLEX\_SUBUNIT\_ORGANIZATION |
| Enrichment Score (ES) | -0.37489802 |
| Normalized Enrichment Score (NES) | -1.7478609 |
| Nominal p-value | 0.0 |
| FDR q-value | 0.115923636 |
| FWER p-Value | 0.999 |
Table: GSEA Results Summary

  

Fig 1: Enrichment plot: GOBP\_RIBONUCLEOPROTEIN\_COMPLEX\_SUBUNIT\_ORGANIZATION      
 Profile of the Running ES Score & Positions of GeneSet Members on the Rank Ordered List

  

| SYMBOL | TITLE | RANK IN GENE LIST | RANK METRIC SCORE | RUNNING ES | CORE ENRICHMENT || 1 | LSM4 | "LSM4 homolog, U6 small nuclear RNA and mRNA degradation associated [Source:HGNC Symbol;Acc:HGNC:17259]" | 174 | 0.837 | 0.0005 | No |
| 2 | TXNL4A | thioredoxin like 4A [Source:HGNC Symbol;Acc:HGNC:30551] | 321 | 0.736 | 0.0016 | No |
| 3 | SRPK3 | SRSF protein kinase 3 [Source:HGNC Symbol;Acc:HGNC:11402] | 329 | 0.734 | 0.0135 | No |
| 4 | MCTS1 | MCTS1 re-initiation and release factor [Source:HGNC Symbol;Acc:HGNC:23357] | 527 | 0.649 | 0.0090 | No |
| 5 | CLP1 | cleavage factor polyribonucleotide kinase subunit 1 [Source:HGNC Symbol;Acc:HGNC:16999] | 748 | 0.582 | 0.0016 | No |
| 6 | CRNKL1 | crooked neck pre-mRNA splicing factor 1 [Source:HGNC Symbol;Acc:HGNC:15762] | 803 | 0.568 | 0.0070 | No |
| 7 | PRPF18 | pre-mRNA processing factor 18 [Source:HGNC Symbol;Acc:HGNC:17351] | 836 | 0.559 | 0.0140 | No |
| 8 | SRSF9 | serine and arginine rich splicing factor 9 [Source:HGNC Symbol;Acc:HGNC:10791] | 882 | 0.548 | 0.0197 | No |
| 9 | DDX23 | DEAD-box helicase 23 [Source:HGNC Symbol;Acc:HGNC:17347] | 913 | 0.538 | 0.0265 | No |
| 10 | USP39 | ubiquitin specific peptidase 39 [Source:HGNC Symbol;Acc:HGNC:20071] | 991 | 0.519 | 0.0293 | No |
| 11 | DHX29 | DExH-box helicase 29 [Source:HGNC Symbol;Acc:HGNC:15815] | 1012 | 0.515 | 0.0364 | No |
| 12 | XRCC5 | X-ray repair cross complementing 5 [Source:HGNC Symbol;Acc:HGNC:12833] | 1018 | 0.513 | 0.0447 | No |
| 13 | SNRPD3 | small nuclear ribonucleoprotein D3 polypeptide [Source:HGNC Symbol;Acc:HGNC:11160] | 1046 | 0.508 | 0.0512 | No |
| 14 | CD2BP2 | CD2 cytoplasmic tail binding protein 2 [Source:HGNC Symbol;Acc:HGNC:1656] | 1158 | 0.485 | 0.0508 | No |
| 15 | EIF2D | eukaryotic translation initiation factor 2D [Source:HGNC Symbol;Acc:HGNC:6583] | 1162 | 0.485 | 0.0587 | No |
| 16 | PRKRA | protein activator of interferon induced protein kinase EIF2AK2 [Source:HGNC Symbol;Acc:HGNC:9438] | 1222 | 0.472 | 0.0621 | No |
| 17 | SNRPD2 | small nuclear ribonucleoprotein D2 polypeptide [Source:HGNC Symbol;Acc:HGNC:11159] | 1290 | 0.460 | 0.0647 | No |
| 18 | MRPS11 | mitochondrial ribosomal protein S11 [Source:HGNC Symbol;Acc:HGNC:14050] | 1305 | 0.457 | 0.0713 | No |
| 19 | GEMIN2 | gem nuclear organelle associated protein 2 [Source:HGNC Symbol;Acc:HGNC:10884] | 1322 | 0.454 | 0.0778 | No |
| 20 | PSIP1 | PC4 and SFRS1 interacting protein 1 [Source:HGNC Symbol;Acc:HGNC:9527] | 1482 | 0.431 | 0.0726 | No |
| 21 | ZNHIT3 | zinc finger HIT-type containing 3 [Source:HGNC Symbol;Acc:HGNC:12309] | 1648 | 0.407 | 0.0665 | No |
| 22 | MRPS7 | mitochondrial ribosomal protein S7 [Source:HGNC Symbol;Acc:HGNC:14499] | 1650 | 0.406 | 0.0734 | No |
| 23 | CLNS1A | chloride nucleotide-sensitive channel 1A [Source:HGNC Symbol;Acc:HGNC:2080] | 1671 | 0.403 | 0.0786 | No |
| 24 | NUDT21 | nudix hydrolase 21 [Source:HGNC Symbol;Acc:HGNC:13870] | 1734 | 0.393 | 0.0804 | No |
| 25 | YJU2 | YJU2 splicing factor homolog [Source:HGNC Symbol;Acc:HGNC:25518] | 1832 | 0.380 | 0.0793 | No |
| 26 | ZFAND1 | zinc finger AN1-type containing 1 [Source:HGNC Symbol;Acc:HGNC:25858] | 1984 | 0.363 | 0.0736 | No |
| 27 | TARBP2 | TARBP2 subunit of RISC loading complex [Source:HGNC Symbol;Acc:HGNC:11569] | 2311 | 0.325 | 0.0535 | No |
| 28 | PIH1D1 | PIH1 domain containing 1 [Source:HGNC Symbol;Acc:HGNC:26075] | 2667 | 0.283 | 0.0304 | No |
| 29 | RUVBL2 | RuvB like AAA ATPase 2 [Source:HGNC Symbol;Acc:HGNC:10475] | 2778 | 0.272 | 0.0264 | No |
| 30 | SF3A1 | splicing factor 3a subunit 1 [Source:HGNC Symbol;Acc:HGNC:10765] | 3152 | 0.235 | 0.0011 | No |
| 31 | GEMIN8 | gem nuclear organelle associated protein 8 [Source:HGNC Symbol;Acc:HGNC:26044] | 3156 | 0.235 | 0.0048 | No |
| 32 | PUF60 | poly(U) binding splicing factor 60 [Source:HGNC Symbol;Acc:HGNC:17042] | 3237 | 0.227 | 0.0024 | No |
| 33 | PRMT7 | protein arginine methyltransferase 7 [Source:HGNC Symbol;Acc:HGNC:25557] | 3250 | 0.226 | 0.0053 | No |
| 34 | DDX1 | DEAD-box helicase 1 [Source:HGNC Symbol;Acc:HGNC:2734] | 3305 | 0.221 | 0.0048 | No |
| 35 | AGO1 | argonaute RISC component 1 [Source:HGNC Symbol;Acc:HGNC:3262] | 3430 | 0.208 | -0.0014 | No |
| 36 | SNRPF | small nuclear ribonucleoprotein polypeptide F [Source:HGNC Symbol;Acc:HGNC:11162] | 3509 | 0.201 | -0.0041 | No |
| 37 | VCP | valosin containing protein [Source:HGNC Symbol;Acc:HGNC:12666] | 3518 | 0.201 | -0.0013 | No |
| 38 | SNRPC | small nuclear ribonucleoprotein polypeptide C [Source:HGNC Symbol;Acc:HGNC:11157] | 3649 | 0.190 | -0.0083 | No |
| 39 | COIL | coilin [Source:HGNC Symbol;Acc:HGNC:2184] | 3684 | 0.187 | -0.0078 | No |
| 40 | GEMIN6 | gem nuclear organelle associated protein 6 [Source:HGNC Symbol;Acc:HGNC:20044] | 3741 | 0.182 | -0.0091 | No |
| 41 | ZRSR2 | "zinc finger CCCH-type, RNA binding motif and serine/arginine rich 2 [Source:HGNC Symbol;Acc:HGNC:23019]" | 3771 | 0.179 | -0.0084 | No |
| 42 | SNU13 | small nuclear ribonucleoprotein 13 [Source:HGNC Symbol;Acc:HGNC:7819] | 3795 | 0.178 | -0.0072 | No |
| 43 | RPS27L | ribosomal protein S27 like [Source:HGNC Symbol;Acc:HGNC:18476] | 3882 | 0.166 | -0.0111 | No |
| 44 | AGO4 | argonaute RISC component 4 [Source:HGNC Symbol;Acc:HGNC:18424] | 4346 | 0.124 | -0.0453 | No |
| 45 | PRPF6 | pre-mRNA processing factor 6 [Source:HGNC Symbol;Acc:HGNC:15860] | 4567 | 0.108 | -0.0608 | No |
| 46 | DDX28 | DEAD-box helicase 28 [Source:HGNC Symbol;Acc:HGNC:17330] | 4602 | 0.105 | -0.0617 | No |
| 47 | LSM2 | "LSM2 homolog, U6 small nuclear RNA and mRNA degradation associated [Source:HGNC Symbol;Acc:HGNC:13940]" | 4641 | 0.101 | -0.0629 | No |
| 48 | ABT1 | activator of basal transcription 1 [Source:HGNC Symbol;Acc:HGNC:17369] | 4783 | 0.088 | -0.0725 | No |
| 49 | EIF2S2 | eukaryotic translation initiation factor 2 subunit beta [Source:HGNC Symbol;Acc:HGNC:3266] | 4838 | 0.084 | -0.0753 | No |
| 50 | EIF4H | eukaryotic translation initiation factor 4H [Source:HGNC Symbol;Acc:HGNC:12741] | 5118 | 0.062 | -0.0962 | No |
| 51 | PRPF8 | pre-mRNA processing factor 8 [Source:HGNC Symbol;Acc:HGNC:17340] | 5287 | 0.049 | -0.1085 | No |
| 52 | STRAP | serine/threonine kinase receptor associated protein [Source:HGNC Symbol;Acc:HGNC:30796] | 5375 | 0.042 | -0.1147 | No |
| 53 | DHX8 | DEAH-box helicase 8 [Source:HGNC Symbol;Acc:HGNC:2749] | 5531 | 0.030 | -0.1263 | No |
| 54 | SNRPE | small nuclear ribonucleoprotein polypeptide E [Source:HGNC Symbol;Acc:HGNC:11161] | 5551 | 0.029 | -0.1273 | No |
| 55 | NOPCHAP1 | NOP protein chaperone 1 [Source:HGNC Symbol;Acc:HGNC:28628] | 5552 | 0.029 | -0.1268 | No |
| 56 | CDC73 | cell division cycle 73 [Source:HGNC Symbol;Acc:HGNC:16783] | 5604 | 0.023 | -0.1304 | No |
| 57 | SART1 | "spliceosome associated factor 1, recruiter of U4/U6.U5 tri-snRNP [Source:HGNC Symbol;Acc:HGNC:10538]" | 5690 | 0.017 | -0.1368 | No |
| 58 | RPS19 | ribosomal protein S19 [Source:HGNC Symbol;Acc:HGNC:10402] | 5952 | -0.002 | -0.1573 | No |
| 59 | MRPL20 | mitochondrial ribosomal protein L20 [Source:HGNC Symbol;Acc:HGNC:14478] | 5989 | -0.004 | -0.1601 | No |
| 60 | SF3A2 | splicing factor 3a subunit 2 [Source:HGNC Symbol;Acc:HGNC:10766] | 5999 | -0.004 | -0.1607 | No |
| 61 | EIF6 | eukaryotic translation initiation factor 6 [Source:HGNC Symbol;Acc:HGNC:6159] | 6140 | -0.015 | -0.1714 | No |
| 62 | ERAL1 | Era like 12S mitochondrial rRNA chaperone 1 [Source:HGNC Symbol;Acc:HGNC:3424] | 6158 | -0.017 | -0.1725 | No |
| 63 | ATM | ATM serine/threonine kinase [Source:HGNC Symbol;Acc:HGNC:795] | 6179 | -0.019 | -0.1737 | No |
| 64 | TAF9 | TATA-box binding protein associated factor 9 [Source:HGNC Symbol;Acc:HGNC:11542] | 6297 | -0.027 | -0.1825 | No |
| 65 | NOP53 | NOP53 ribosome biogenesis factor [Source:HGNC Symbol;Acc:HGNC:4333] | 6334 | -0.031 | -0.1848 | No |
| 66 | AAR2 | AAR2 splicing factor [Source:HGNC Symbol;Acc:HGNC:15886] | 6401 | -0.035 | -0.1893 | No |
| 67 | RPL10L | ribosomal protein L10 like [Source:HGNC Symbol;Acc:HGNC:17976] | 6502 | -0.042 | -0.1965 | No |
| 68 | DENR | density regulated re-initiation and release factor [Source:HGNC Symbol;Acc:HGNC:2769] | 6611 | -0.050 | -0.2041 | No |
| 69 | EIF2S3 | eukaryotic translation initiation factor 2 subunit gamma [Source:HGNC Symbol;Acc:HGNC:3267] | 6870 | -0.069 | -0.2232 | No |
| 70 | EIF5 | eukaryotic translation initiation factor 5 [Source:HGNC Symbol;Acc:HGNC:3299] | 6934 | -0.074 | -0.2269 | No |
| 71 | SNRNP200 | small nuclear ribonucleoprotein U5 subunit 200 [Source:HGNC Symbol;Acc:HGNC:30859] | 7249 | -0.099 | -0.2498 | No |
| 72 | NLE1 | notchless homolog 1 [Source:HGNC Symbol;Acc:HGNC:19889] | 7253 | -0.099 | -0.2484 | No |
| 73 | KLC1 | kinesin light chain 1 [Source:HGNC Symbol;Acc:HGNC:6387] | 7343 | -0.105 | -0.2536 | No |
| 74 | RUVBL1 | RuvB like AAA ATPase 1 [Source:HGNC Symbol;Acc:HGNC:10474] | 7477 | -0.116 | -0.2621 | No |
| 75 | NOL3 | nucleolar protein 3 [Source:HGNC Symbol;Acc:HGNC:7869] | 7545 | -0.122 | -0.2653 | No |
| 76 | POLR2D | RNA polymerase II subunit D [Source:HGNC Symbol;Acc:HGNC:9191] | 7726 | -0.136 | -0.2771 | No |
| 77 | FASTKD2 | FAST kinase domains 2 [Source:HGNC Symbol;Acc:HGNC:29160] | 7730 | -0.136 | -0.2750 | No |
| 78 | TGS1 | trimethylguanosine synthase 1 [Source:HGNC Symbol;Acc:HGNC:17843] | 7791 | -0.141 | -0.2773 | No |
| 79 | CPSF6 | cleavage and polyadenylation specific factor 6 [Source:HGNC Symbol;Acc:HGNC:13871] | 7885 | -0.147 | -0.2822 | No |
| 80 | PRPF31 | pre-mRNA processing factor 31 [Source:HGNC Symbol;Acc:HGNC:15446] | 7898 | -0.147 | -0.2806 | No |
| 81 | PRMT5 | protein arginine methyltransferase 5 [Source:HGNC Symbol;Acc:HGNC:10894] | 7904 | -0.148 | -0.2785 | No |
| 82 | EIF4B | eukaryotic translation initiation factor 4B [Source:HGNC Symbol;Acc:HGNC:3285] | 8051 | -0.159 | -0.2872 | No |
| 83 | RPL3L | ribosomal protein L3 like [Source:HGNC Symbol;Acc:HGNC:10351] | 8097 | -0.163 | -0.2880 | No |
| 84 | DHX30 | DExH-box helicase 30 [Source:HGNC Symbol;Acc:HGNC:16716] | 8111 | -0.164 | -0.2863 | No |
| 85 | SETX | senataxin [Source:HGNC Symbol;Acc:HGNC:445] | 8398 | -0.185 | -0.3056 | No |
| 86 | XAB2 | XPA binding protein 2 [Source:HGNC Symbol;Acc:HGNC:14089] | 8778 | -0.215 | -0.3317 | No |
| 87 | PTGES3 | prostaglandin E synthase 3 [Source:HGNC Symbol;Acc:HGNC:16049] | 8802 | -0.217 | -0.3298 | No |
| 88 | RPSA | ribosomal protein SA [Source:HGNC Symbol;Acc:HGNC:6502] | 8866 | -0.222 | -0.3310 | No |
| 89 | SNRPG | small nuclear ribonucleoprotein polypeptide G [Source:HGNC Symbol;Acc:HGNC:11163] | 9067 | -0.237 | -0.3427 | No |
| 90 | SNRPB | small nuclear ribonucleoprotein polypeptides B and B1 [Source:HGNC Symbol;Acc:HGNC:11153] | 9085 | -0.239 | -0.3400 | No |
| 91 | TFIP11 | tuftelin interacting protein 11 [Source:HGNC Symbol;Acc:HGNC:17165] | 9098 | -0.239 | -0.3368 | No |
| 92 | USP4 | ubiquitin specific peptidase 4 [Source:HGNC Symbol;Acc:HGNC:12627] | 9130 | -0.242 | -0.3352 | No |
| 93 | NCBP1 | nuclear cap binding protein subunit 1 [Source:HGNC Symbol;Acc:HGNC:7658] | 9149 | -0.244 | -0.3324 | No |
| 94 | ADAR | adenosine deaminase RNA specific [Source:HGNC Symbol;Acc:HGNC:225] | 9195 | -0.247 | -0.3318 | No |
| 95 | GEMIN7 | gem nuclear organelle associated protein 7 [Source:HGNC Symbol;Acc:HGNC:20045] | 9441 | -0.268 | -0.3465 | No |
| 96 | SNRPD1 | small nuclear ribonucleoprotein D1 polypeptide [Source:HGNC Symbol;Acc:HGNC:11158] | 9579 | -0.281 | -0.3525 | No |
| 97 | PRKDC | "protein kinase, DNA-activated, catalytic subunit [Source:HGNC Symbol;Acc:HGNC:9413]" | 9632 | -0.286 | -0.3517 | No |
| 98 | GCFC2 | GC-rich sequence DNA-binding factor 2 [Source:HGNC Symbol;Acc:HGNC:1317] | 9908 | -0.312 | -0.3680 | No |
| 99 | KHDC4 | "KH domain containing 4, pre-mRNA splicing factor [Source:HGNC Symbol;Acc:HGNC:29145]" | 9997 | -0.321 | -0.3695 | Yes |
| 100 | SHQ1 | "SHQ1, H/ACA ribonucleoprotein assembly factor [Source:HGNC Symbol;Acc:HGNC:25543]" | 10045 | -0.326 | -0.3676 | Yes |
| 101 | HSP90AB1 | heat shock protein 90 alpha family class B member 1 [Source:HGNC Symbol;Acc:HGNC:5258] | 10060 | -0.327 | -0.3632 | Yes |
| 102 | GEMIN4 | gem nuclear organelle associated protein 4 [Source:HGNC Symbol;Acc:HGNC:15717] | 10075 | -0.328 | -0.3587 | Yes |
| 103 | RPL5 | ribosomal protein L5 [Source:HGNC Symbol;Acc:HGNC:10360] | 10201 | -0.341 | -0.3627 | Yes |
| 104 | ZNHIT6 | zinc finger HIT-type containing 6 [Source:HGNC Symbol;Acc:HGNC:26089] | 10220 | -0.343 | -0.3583 | Yes |
| 105 | RPL24 | ribosomal protein L24 [Source:HGNC Symbol;Acc:HGNC:10325] | 10263 | -0.347 | -0.3557 | Yes |
| 106 | SRSF1 | serine and arginine rich splicing factor 1 [Source:HGNC Symbol;Acc:HGNC:10780] | 10272 | -0.348 | -0.3505 | Yes |
| 107 | BRIX1 | biogenesis of ribosomes BRX1 [Source:HGNC Symbol;Acc:HGNC:24170] | 10275 | -0.348 | -0.3447 | Yes |
| 108 | SRSF5 | serine and arginine rich splicing factor 5 [Source:HGNC Symbol;Acc:HGNC:10787] | 10282 | -0.349 | -0.3393 | Yes |
| 109 | MRTO4 | "MRT4 homolog, ribosome maturation factor [Source:HGNC Symbol;Acc:HGNC:18477]" | 10300 | -0.350 | -0.3347 | Yes |
| 110 | SF3A3 | splicing factor 3a subunit 3 [Source:HGNC Symbol;Acc:HGNC:10767] | 10311 | -0.352 | -0.3295 | Yes |
| 111 | LUC7L2 | "LUC7 like 2, pre-mRNA splicing factor [Source:HGNC Symbol;Acc:HGNC:21608]" | 10334 | -0.354 | -0.3252 | Yes |
| 112 | SCAF11 | SR-related CTD associated factor 11 [Source:HGNC Symbol;Acc:HGNC:10784] | 10375 | -0.358 | -0.3223 | Yes |
| 113 | RRP7A | ribosomal RNA processing 7 homolog A [Source:HGNC Symbol;Acc:HGNC:24286] | 10449 | -0.367 | -0.3218 | Yes |
| 114 | SRPK2 | SRSF protein kinase 2 [Source:HGNC Symbol;Acc:HGNC:11306] | 10522 | -0.376 | -0.3210 | Yes |
| 115 | RPS14 | ribosomal protein S14 [Source:HGNC Symbol;Acc:HGNC:10387] | 10558 | -0.379 | -0.3174 | Yes |
| 116 | DHX9 | DExH-box helicase 9 [Source:HGNC Symbol;Acc:HGNC:2750] | 10627 | -0.386 | -0.3162 | Yes |
| 117 | LUC7L3 | LUC7 like 3 pre-mRNA splicing factor [Source:HGNC Symbol;Acc:HGNC:24309] | 10647 | -0.388 | -0.3111 | Yes |
| 118 | SRSF6 | serine and arginine rich splicing factor 6 [Source:HGNC Symbol;Acc:HGNC:10788] | 10659 | -0.389 | -0.3053 | Yes |
| 119 | RPL10 | ribosomal protein L10 [Source:HGNC Symbol;Acc:HGNC:10298] | 10684 | -0.391 | -0.3006 | Yes |
| 120 | YTHDC1 | YTH domain containing 1 [Source:HGNC Symbol;Acc:HGNC:30626] | 10687 | -0.391 | -0.2941 | Yes |
| 121 | CELF2 | CUGBP Elav-like family member 2 [Source:HGNC Symbol;Acc:HGNC:2550] | 10832 | -0.409 | -0.2985 | Yes |
| 122 | RBM5 | RNA binding motif protein 5 [Source:HGNC Symbol;Acc:HGNC:9902] | 10849 | -0.410 | -0.2928 | Yes |
| 123 | SRSF10 | serine and arginine rich splicing factor 10 [Source:HGNC Symbol;Acc:HGNC:16713] | 10926 | -0.422 | -0.2916 | Yes |
| 124 | SF3B1 | splicing factor 3b subunit 1 [Source:HGNC Symbol;Acc:HGNC:10768] | 10948 | -0.424 | -0.2860 | Yes |
| 125 | RRS1 | ribosome biogenesis regulator 1 homolog [Source:HGNC Symbol;Acc:HGNC:17083] | 11045 | -0.437 | -0.2862 | Yes |
| 126 | RPL38 | ribosomal protein L38 [Source:HGNC Symbol;Acc:HGNC:10349] | 11122 | -0.446 | -0.2846 | Yes |
| 127 | RPL6 | ribosomal protein L6 [Source:HGNC Symbol;Acc:HGNC:10362] | 11129 | -0.447 | -0.2775 | Yes |
| 128 | RPS5 | ribosomal protein S5 [Source:HGNC Symbol;Acc:HGNC:10426] | 11206 | -0.458 | -0.2757 | Yes |
| 129 | ATR | ATR serine/threonine kinase [Source:HGNC Symbol;Acc:HGNC:882] | 11231 | -0.461 | -0.2697 | Yes |
| 130 | AGO2 | argonaute RISC catalytic component 2 [Source:HGNC Symbol;Acc:HGNC:3263] | 11246 | -0.463 | -0.2630 | Yes |
| 131 | RPL11 | ribosomal protein L11 [Source:HGNC Symbol;Acc:HGNC:10301] | 11252 | -0.464 | -0.2555 | Yes |
| 132 | PRPF19 | pre-mRNA processing factor 19 [Source:HGNC Symbol;Acc:HGNC:17896] | 11269 | -0.467 | -0.2488 | Yes |
| 133 | CELF3 | CUGBP Elav-like family member 3 [Source:HGNC Symbol;Acc:HGNC:11967] | 11510 | -0.502 | -0.2592 | Yes |
| 134 | PWP2 | PWP2 small subunit processome component [Source:HGNC Symbol;Acc:HGNC:9711] | 11604 | -0.516 | -0.2577 | Yes |
| 135 | NUFIP1 | nuclear FMR1 interacting protein 1 [Source:HGNC Symbol;Acc:HGNC:8057] | 11614 | -0.519 | -0.2496 | Yes |
| 136 | LUC7L | LUC7 like [Source:HGNC Symbol;Acc:HGNC:6723] | 11627 | -0.521 | -0.2417 | Yes |
| 137 | SF1 | splicing factor 1 [Source:HGNC Symbol;Acc:HGNC:12950] | 11664 | -0.527 | -0.2356 | Yes |
| 138 | RPS15 | ribosomal protein S15 [Source:HGNC Symbol;Acc:HGNC:10388] | 11671 | -0.527 | -0.2271 | Yes |
| 139 | PRPF39 | pre-mRNA processing factor 39 [Source:HGNC Symbol;Acc:HGNC:20314] | 11854 | -0.560 | -0.2319 | Yes |
| 140 | SRPK1 | SRSF protein kinase 1 [Source:HGNC Symbol;Acc:HGNC:11305] | 11889 | -0.569 | -0.2249 | Yes |
| 141 | MDN1 | midasin AAA ATPase 1 [Source:HGNC Symbol;Acc:HGNC:18302] | 11973 | -0.588 | -0.2215 | Yes |
| 142 | PPAN | peter pan homolog [Source:HGNC Symbol;Acc:HGNC:9227] | 12022 | -0.597 | -0.2151 | Yes |
| 143 | RPS27 | ribosomal protein S27 [Source:HGNC Symbol;Acc:HGNC:10416] | 12071 | -0.612 | -0.2085 | Yes |
| 144 | SMN1 | "survival of motor neuron 1, telomeric [Source:HGNC Symbol;Acc:HGNC:11117]" | 12102 | -0.621 | -0.2003 | Yes |
| 145 | SART3 | "spliceosome associated factor 3, U4/U6 recycling protein [Source:HGNC Symbol;Acc:HGNC:16860]" | 12161 | -0.633 | -0.1941 | Yes |
| 146 | RPL13A | ribosomal protein L13a [Source:HGNC Symbol;Acc:HGNC:10304] | 12200 | -0.645 | -0.1862 | Yes |
| 147 | PTBP2 | polypyrimidine tract binding protein 2 [Source:HGNC Symbol;Acc:HGNC:17662] | 12211 | -0.649 | -0.1759 | Yes |
| 148 | DICER1 | "dicer 1, ribonuclease III [Source:HGNC Symbol;Acc:HGNC:17098]" | 12215 | -0.650 | -0.1651 | Yes |
| 149 | CPSF7 | cleavage and polyadenylation specific factor 7 [Source:HGNC Symbol;Acc:HGNC:30098] | 12251 | -0.661 | -0.1567 | Yes |
| 150 | RPLP0 | ribosomal protein lateral stalk subunit P0 [Source:HGNC Symbol;Acc:HGNC:10371] | 12285 | -0.672 | -0.1479 | Yes |
| 151 | RPL3 | ribosomal protein L3 [Source:HGNC Symbol;Acc:HGNC:10332] | 12350 | -0.691 | -0.1412 | Yes |
| 152 | NOP2 | NOP2 nucleolar protein [Source:HGNC Symbol;Acc:HGNC:7867] | 12372 | -0.699 | -0.1310 | Yes |
| 153 | DYRK3 | dual specificity tyrosine phosphorylation regulated kinase 3 [Source:HGNC Symbol;Acc:HGNC:3094] | 12379 | -0.702 | -0.1195 | Yes |
| 154 | CELF1 | CUGBP Elav-like family member 1 [Source:HGNC Symbol;Acc:HGNC:2549] | 12391 | -0.704 | -0.1084 | Yes |
| 155 | EIF3A | eukaryotic translation initiation factor 3 subunit A [Source:HGNC Symbol;Acc:HGNC:3271] | 12402 | -0.707 | -0.0972 | Yes |
| 156 | RPS28 | ribosomal protein S28 [Source:HGNC Symbol;Acc:HGNC:10418] | 12526 | -0.761 | -0.0940 | Yes |
| 157 | BOP1 | BOP1 ribosomal biogenesis factor [Source:HGNC Symbol;Acc:HGNC:15519] | 12580 | -0.785 | -0.0848 | Yes |
| 158 | KIF5B | kinesin family member 5B [Source:HGNC Symbol;Acc:HGNC:6324] | 12662 | -0.835 | -0.0770 | Yes |
| 159 | AGO3 | argonaute RISC catalytic component 3 [Source:HGNC Symbol;Acc:HGNC:18421] | 12726 | -0.888 | -0.0669 | Yes |
| 160 | HSP90AA1 | heat shock protein 90 alpha family class A member 1 [Source:HGNC Symbol;Acc:HGNC:5253] | 12730 | -0.893 | -0.0520 | Yes |
| 161 | SFSWAP | splicing factor SWAP [Source:HGNC Symbol;Acc:HGNC:10790] | 12747 | -0.908 | -0.0378 | Yes |
| 162 | DDX39B | DExD-box helicase 39B [Source:HGNC Symbol;Acc:HGNC:13917] | 12783 | -0.941 | -0.0246 | Yes |
| 163 | RPL23A | ribosomal protein L23a [Source:HGNC Symbol;Acc:HGNC:10317] | 12785 | -0.944 | -0.0087 | Yes |
| 164 | PRPF3 | pre-mRNA processing factor 3 [Source:HGNC Symbol;Acc:HGNC:17348] | 12837 | -1.040 | 0.0049 | Yes |
Table: GSEA details [plain text format]

  

Fig 2: GOBP\_RIBONUCLEOPROTEIN\_COMPLEX\_SUBUNIT\_ORGANIZATION      
 Blue-Pink O' Gram in the Space of the Analyzed GeneSet

  

Fig 3: GOBP\_RIBONUCLEOPROTEIN\_COMPLEX\_SUBUNIT\_ORGANIZATION: Random ES distribution      
 Gene set null distribution of ES for **GOBP\_RIBONUCLEOPROTEIN\_COMPLEX\_SUBUNIT\_ORGANIZATION**
